# *N*-Methyl-2-{3-methyl-2-[(2*Z*)-pent-2-en-1-yl]cyclo­pent-2-en-1-yl­idene}hydrazinecarbo­thio­amide

**DOI:** 10.1107/S2414314624000130

**Published:** 2024-01-09

**Authors:** Adriano Bof de Oliveira, Leandro Bresolin, Johannes Beck, Jörg Daniels

**Affiliations:** a Departamento de Química, Universidade Federal de Sergipe, Av. Marcelo Deda Chagas s/n, Campus Universitário, 49107-230 São Cristóvão-SE, Brazil; b Escola de Química e Alimentos, Universidade Federal do Rio Grande, Av. Itália km 08, Campus Carreiros, 96203-900 Rio Grande-RS, Brazil; c Institut für Anorganische Chemie, Rheinische Friedrich-Wilhelms-Universität Bonn, Gerhard-Domagk-Strasse 1, D-53121 Bonn, Germany; Goethe-Universität Frankfurt, Germany

**Keywords:** thio­semicarbazone, *cis*-jasmone, methyl­thio­semicarbazone derivative, centrosymmetric dimers, crystal structure, Hirshfeld analysis

## Abstract

The equimolar and hydro­chloric acid-catalysed reaction between *cis*-jasmone and 4-methyl­thio­semicarbazide in ethano­lic solution yields the title compound, C_13_H_21_N_3_S (common name: *cis*-jasmone 4-methyl­thio­semicarbazone). Two mol­ecules with all atoms in general positions are present in the asymmetric unit. In one of them, the carbon chain is disordered [site occupancy ratio = 0.821 (3):0.179 (3)]. The thio­semicarbazone entities [N—N—C(=S)—N] are approximately planar, with the maximum deviation from the mean plane through the selected atoms being −0.0115 (16) Å (r.m.s.d. = 0.0078 Å) for the non-disordered mol­ecule and 0.0052 (14) Å (r.m.s.d. = 0.0031 Å) for the disordered one. The mol­ecules are not planar, since the jasmone groups have a chain with *sp* ^3^-hybridized carbon atoms and, in addition, the thio­semicarbazone fragments are attached to the respective carbon five-membered rings and the dihedral angles between them for each mol­ecule amount to 8.9 (1) and 6.3 (1)°. In the crystal, the mol­ecules are connected through pairs of N—H⋯S and C—H⋯S inter­actions into crystallographically independent centrosymmetric dimers, in which rings of graph-set motifs *R*_2_^2^(8) and *R*_2_^1^(7) are observed. A Hirshfeld surface analysis indicates that the major contributions for the crystal cohesion are from H⋯H (70.6%), H⋯S/S⋯H (16.7%), H⋯C/C⋯H (7.5%) and H⋯N/N⋯H (4.9%) inter­actions [considering the two crystallographically independent mol­ecules and only the disordered atoms with the highest s.o.f. for the evaluation].

## Abstract 2

The synthesis, crystal structure and Hirshfeld analysis of *cis*-jasmone 4-methyl­thio­semicarbazone is reported. Two crystallographically independent mol­ecules are observed in the asymmetric unit, one of them being disordered over the carbon chain. In the crystal, the mol­ecules are linked by N—H⋯S and C—H⋯S inter­actions into independent centrosymmetric dimers.

## Graphical abstract

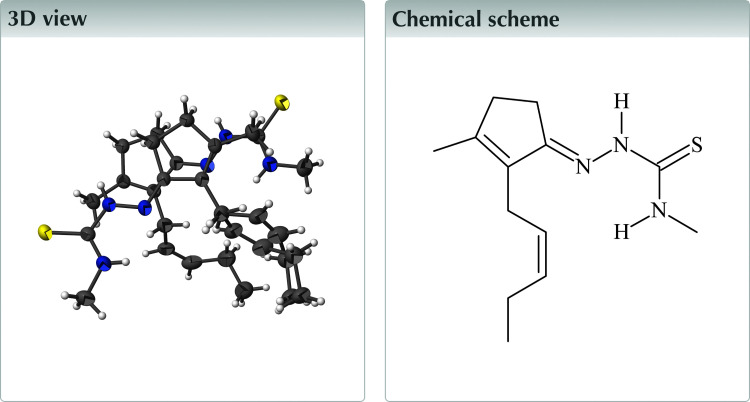

## Structure description

To the best of our knowledge, the first crystal structure of *cis*-jasmone thio­semicarbazone was reported recently and it was pointed out that this derivative based on non-substituted *cis*-jasmone shows anti­fungal activity (Orsoni *et al.*, 2020 ▸; Jamiołkowska *et al.*, 2022 ▸).

As part of our inter­est in thio­semicarbazones attached to natural product derivatives and on the influence of the substituent groups at the terminal N atom on the supra­molecular arrangement, we report here the synthesis, crystal structure and Hirshfeld analysis of *cis*-jasmone 4-methyl­thio­semicarbazone. It is important to highlight that the substit­uents at the terminal N atom are relevant not only to the crystal packing, but also to the biological properties of the thio­semicarbazone derivatives. For example, a small chemical library of 4-methyl­thio­semicarbazones has been studied for the treatment of Parkinson’s disease (Mathew *et al.*, 2021 ▸) and for microbial growth inhibition (D’Agostino *et al.*, 2022 ▸). In addition, for a review article on coordination compounds with 4-methyl­thio­semicarbazone derivatives including biological applications and catalytic activity, see: Monsur Showkot Hossain *et al.* (2023 ▸).

**Figure 1:**
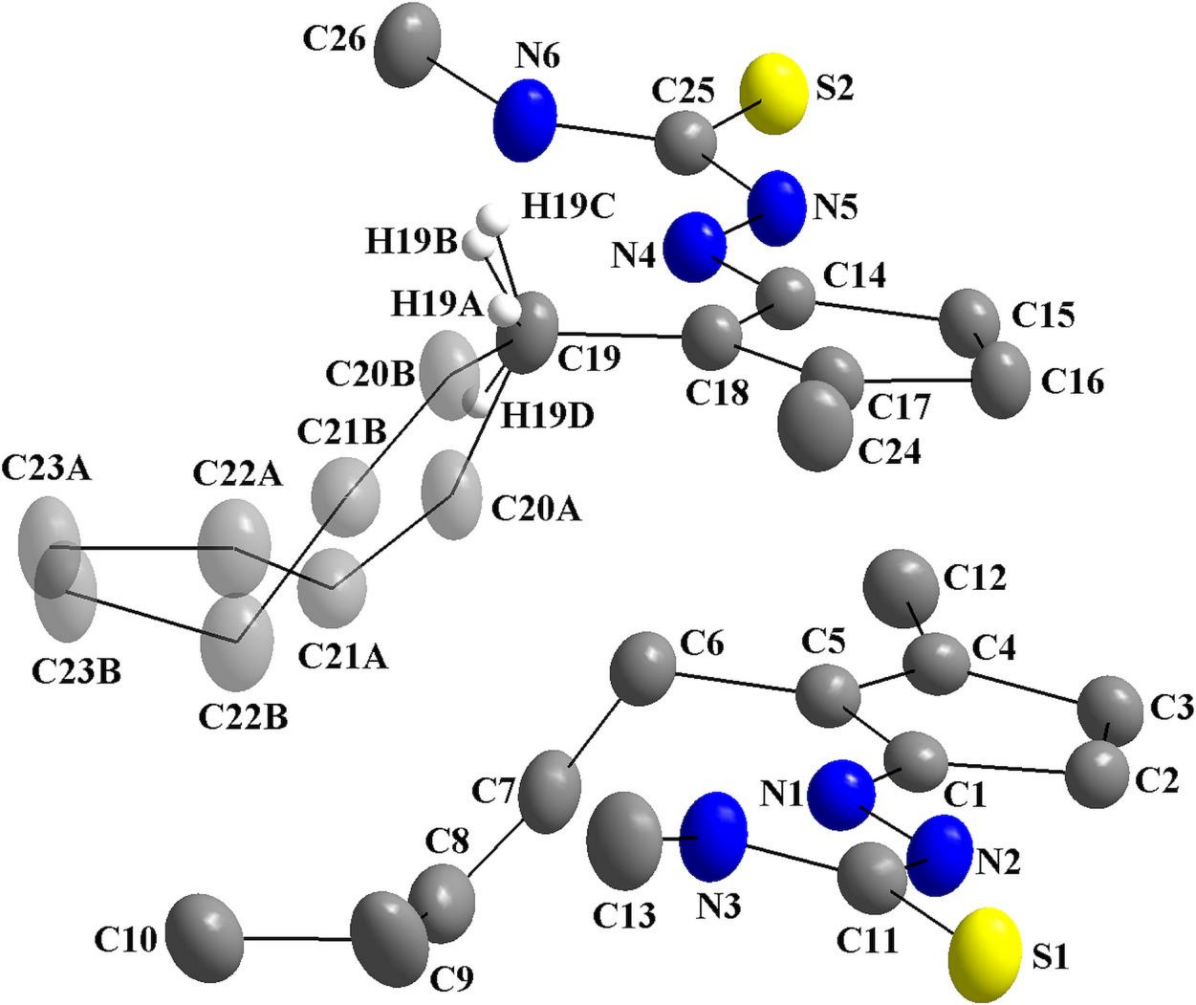
The mol­ecular structure of the title compound, showing the atom labelling and displacement ellipsoids drawn at the 40% probability level for the two crystallographically independent mol­ecules. Disordered atoms are drawn with 40% transparency and labelled C20*A*, C21*A*, C22*A*, C23*A*, H19*A* and H19*B* [s.o.f. = 0.821 (3)] and C20*B*, C21*B*, C22*B*, C23*B*, H19*C* and H19*D* [s.o.f. = 0.179 (3)]. The remaining H atoms were omitted for clarity.

The asymmetric unit of the title compound comprises two mol­ecules with all atoms in general positions, with one of them showing disorder over the carbon chain [site occupancy ratio = 0.821 (3):0.179 (3)]. The mol­ecules are not planar due to the chain with *sp* ^3^-hybridized carbon atoms in the jasmone fragment and the dihedral angles between the thio­semicarbazone fragment and the respective carbon five-membered ring, which amount to 8.9 (1)° for the non-disordered mol­ecule and 6.3 (1)° for the disordered one (Fig. 1 ▸). To simplify the structure description, the non-disordered mol­ecule, with atoms C1–C13/N–N3/S1, will be designated as **JMTSC-1**, while the disordered one, with the atoms C14–C23*A*/C23*B*/N4–N6/S2, will be designated as **JMTSC-2**. To get a stable refinement, the C20, C21, C22 and C23 atoms were split into two positions and *A*-labelled for the higher s.o.f and *B*-labelled for the lower. Atom C19, which is itself not disordered, is bound to C20*A* and C20*B*, and to achieve the best orientations for the C19—H bonds, the H19*A* and H19*B* atoms were also split, into two positions. Thus, the H19*A* and H19*B* atoms have a s.o.f. of 0.821 (3) and the H19*C* and H19*D* atoms have a s.o.f. of 0.179 (3). Selected geometric parameters for the structural description of **JMTSC-1** and **JMTSC-2** are given in Table 1 ▸; these are in agreement with literature data (Oliveira *et al.*, 2016 ▸; Rocha *et al.*, 2014 ▸).

For the supra­molecular arrangement and Hirshfeld analysis, for clarity only the disordered atoms with the highest s.o.f. value were considered. In the crystal, the mol­ecules are connected through pairs of N—H⋯S and C—H⋯S inter­actions into centrosymmetric dimers with graph-set motifs 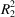 (8) and 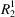 (7) (Table 2 ▸).

**Figure 2:**
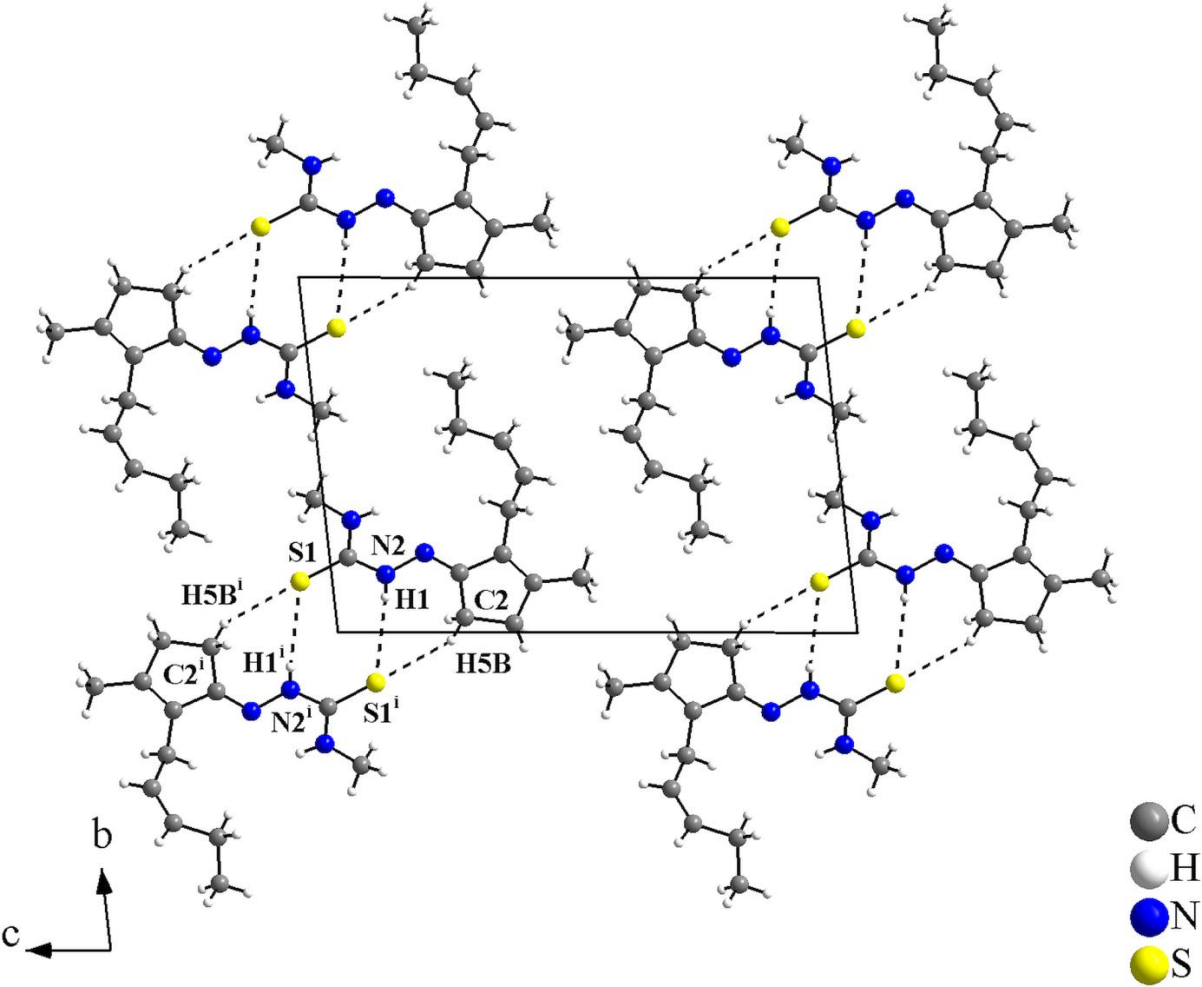
Crystal structure section of the title compound for the **JMTSC-1** mol­ecule, showing the hydrogen-bond inter­molecular inter­actions as dashed lines. The mol­ecules are linked into centrosymmetric dimers *via* pairs of N—H⋯S and C—H⋯S inter­actions with graph-set motifs 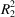 (8) and 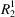 (7). [Symmetry code: (i) −*x*, −*y*, −*z* + 2.]

**Figure 3:**
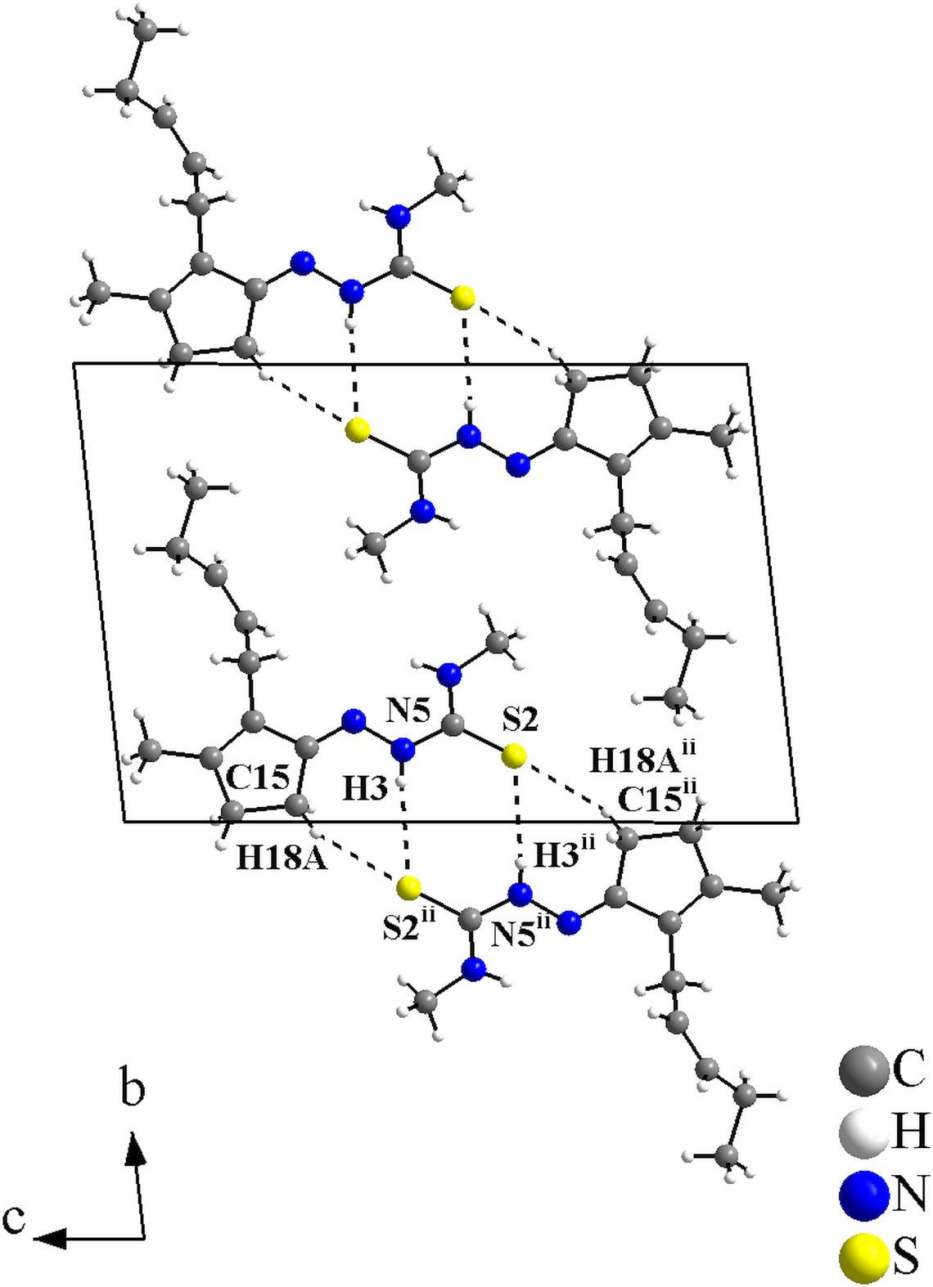
Crystal structure section of the title compound for the **JMTSC-2** mol­ecule, showing the hydrogen-bonded inter­molecular inter­actions drawn as dashed lines. Disorder is not shown for clarity. The mol­ecules are linked into centrosymmetric dimers *via* pairs of N—H⋯S and C—H⋯S inter­actions with graph-set motifs 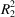 (8) and 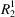 (7). [Symmetry code: (ii) −*x* + 1, −*y*, −*z* + 1.]

**Figure 4:**
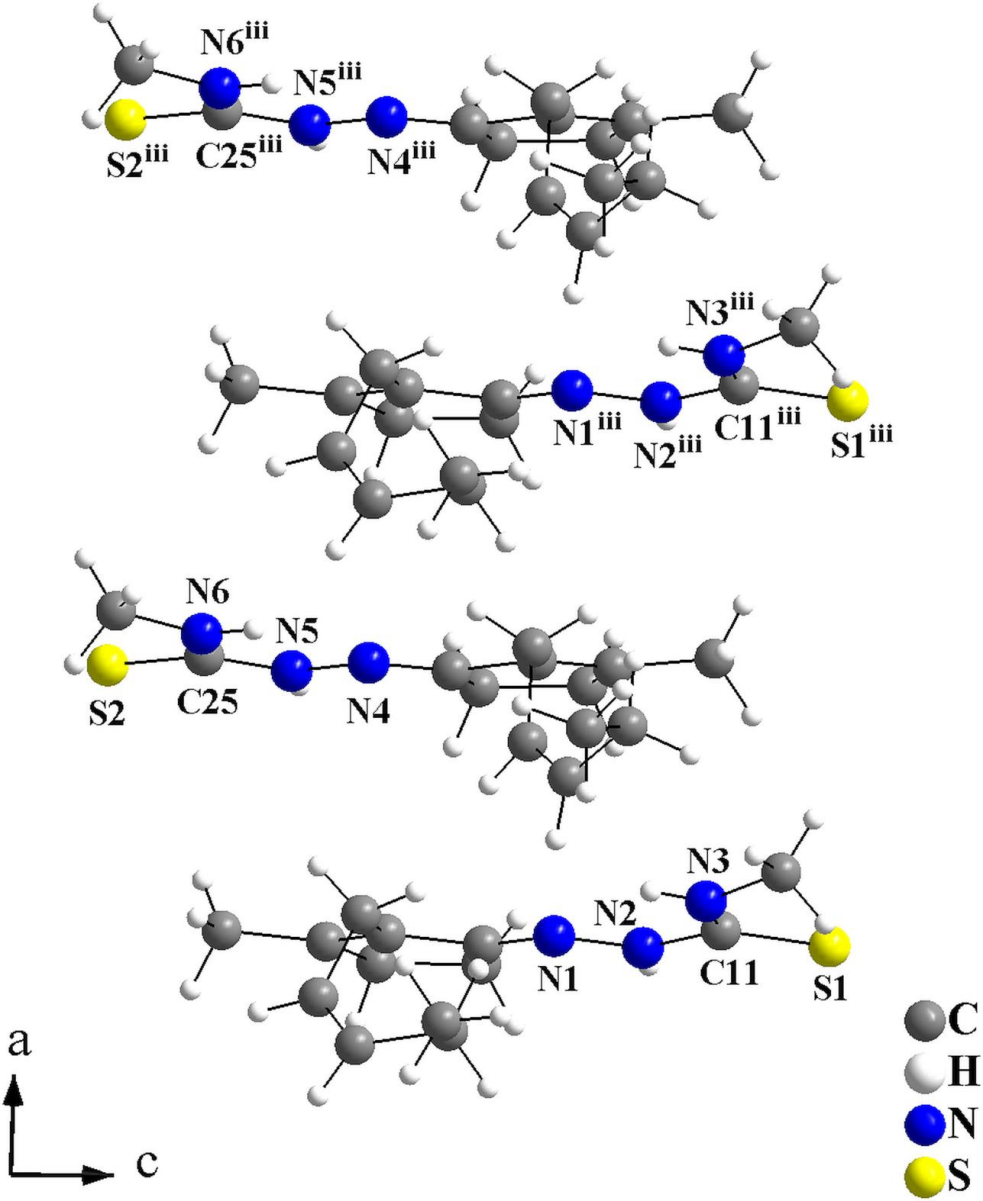
Selected crystal section of the title compound viewed along [010] showing the **JMTSC-1** and **JMTSC-2** mol­ecules stacked along [100]. Only the non-H atoms of the thio­semicarbazone entities are labelled and disorder is not shown for clarity. [Symmetry code: (iii) *x* + 1, *y*, *z*.]

With the coordinates that were used for the refinement, the crystallographically independent dimers of the **JMTSC-1** mol­ecules have the gravity centre located in the cell vertices (Fig. 2 ▸), and in the centre of the *ac* planes for the **JMTSC-2** mol­ecules (Fig. 3 ▸). In addition, the mol­ecules are stacked along [100] and only weak inter­molecular inter­actions, *e.g.*, London dispersion forces can be presumed in this direction (Fig. 4 ▸).

**Figure 5:**
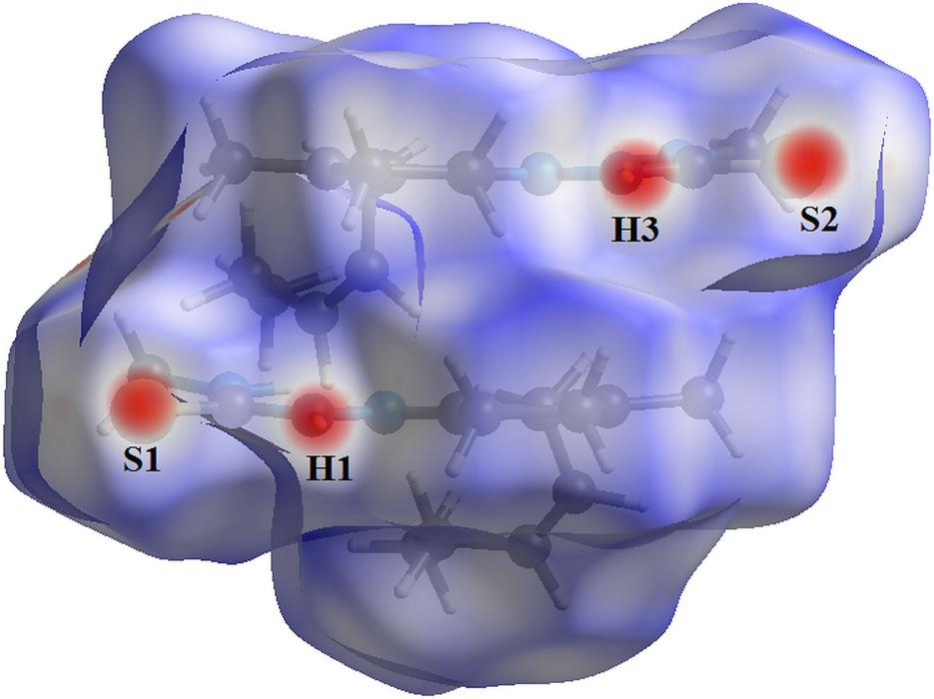
Hirshfeld surface graphical representation (*d*_norm_) for the two crystallographically independent mol­ecules of the title compound. The surface is drawn with transparency, and the disorder is not shown for clarity. The regions with strongest inter­molecular inter­actions are shown in red (*d*_norm_ range: −0.216 to 1.522 a.u.).

**Figure 6:**
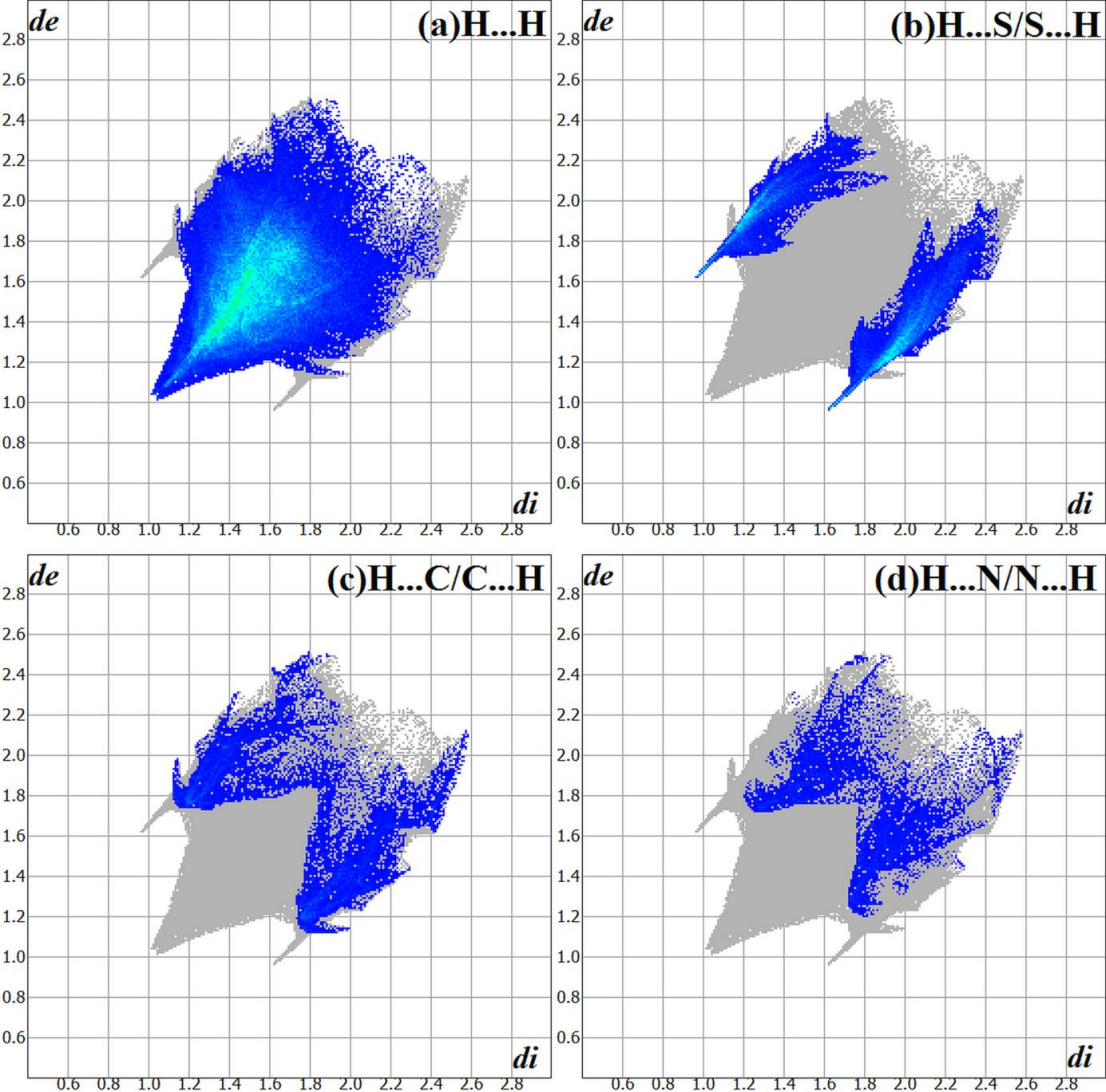
The Hirshfeld surface two-dimensional fingerprint plot for the title compound, showing the contacts in detail (cyan dots). The major contributions of the inter­actions to the crystal cohesion amount to (*a*) H⋯H (70.6%), (*b*) H⋯S/S⋯H (16.7%), (*c*) H⋯C/C⋯H (7.5%) and (*d*) H⋯N/N⋯H (4.9%). The *d*_i_ (*x-*axis) and the *d*_e_ (*y-*axis) values are the closest inter­nal and external distances from given points on the Hirshfeld surface contacts (in Å). Regarding the disorder, only the atoms with the highest s.o.f. were considered.

The Hirshfeld surface analysis (Hirshfeld, 1977 ▸), the graphical representations and the two-dimensional Hirshfeld surface fingerprints (HSFP) were evaluated with the *Crystal Explorer* software (Wolff *et al.*, 2012 ▸). The Hirshfeld surface analysis of the title compound, considering the **JMTSC-1** and the **JMTSC-2** mol­ecules, suggests that the most relevant inter­molecular inter­actions for the crystal packing are H⋯H (70.6%), H⋯S/S⋯H (16.7%), H⋯C/C⋯H (7.5%) and H⋯N/N⋯H (4.9%). A graphical representation of the Hirshfeld surface (*d*_norm_) is shown in Fig. 5 ▸ with the locations of the strongest inter­molecular contacts, *i.e*, the regions around the atoms H1, H3, S1 and S2, indicated in red. These atoms are those involved in the H⋯S inter­actions showed in the previous figures (Figs. 2 ▸ and 3 ▸). The contributions to the crystal cohesion are shown as two-dimensional Hirshfeld surface fingerprint plots (HSFP) with cyan dots (Fig. 6 ▸).

The crystalline supra­molecular arrangement of thio­semicarbazones depends on the template effect of the crystallization solvent, the presence of solvate mol­ecules and on the crystallization methods. In addition, the steric effect of the substituents in the *R*_1_ *R*_2_N—N(H)—C(=S)—N*R*_3_ *R*_4_ fragment is of prime importance for the crystal packing. In the title compound, two structural features lead to the building of dimers. The first one is the terminal methyl group, N(H)CH_3_, which decreases the possibility for N—H⋯S inter­molecular inter­actions and enhances the formation of hydrogen-bonded supra­molecular structures. On the other side of the mol­ecule, the second feature is the *cis*-jasmone entity, which, through steric hindrance, precludes inter­molecular inter­actions, *e.g.*, N—H⋯S or N—H⋯N (Figs. 2 ▸ and 3 ▸); thus, four methyl-substituted thio­semicarbazone derivatives were selected for structural comparison with the title compound.

**Figure 7:**
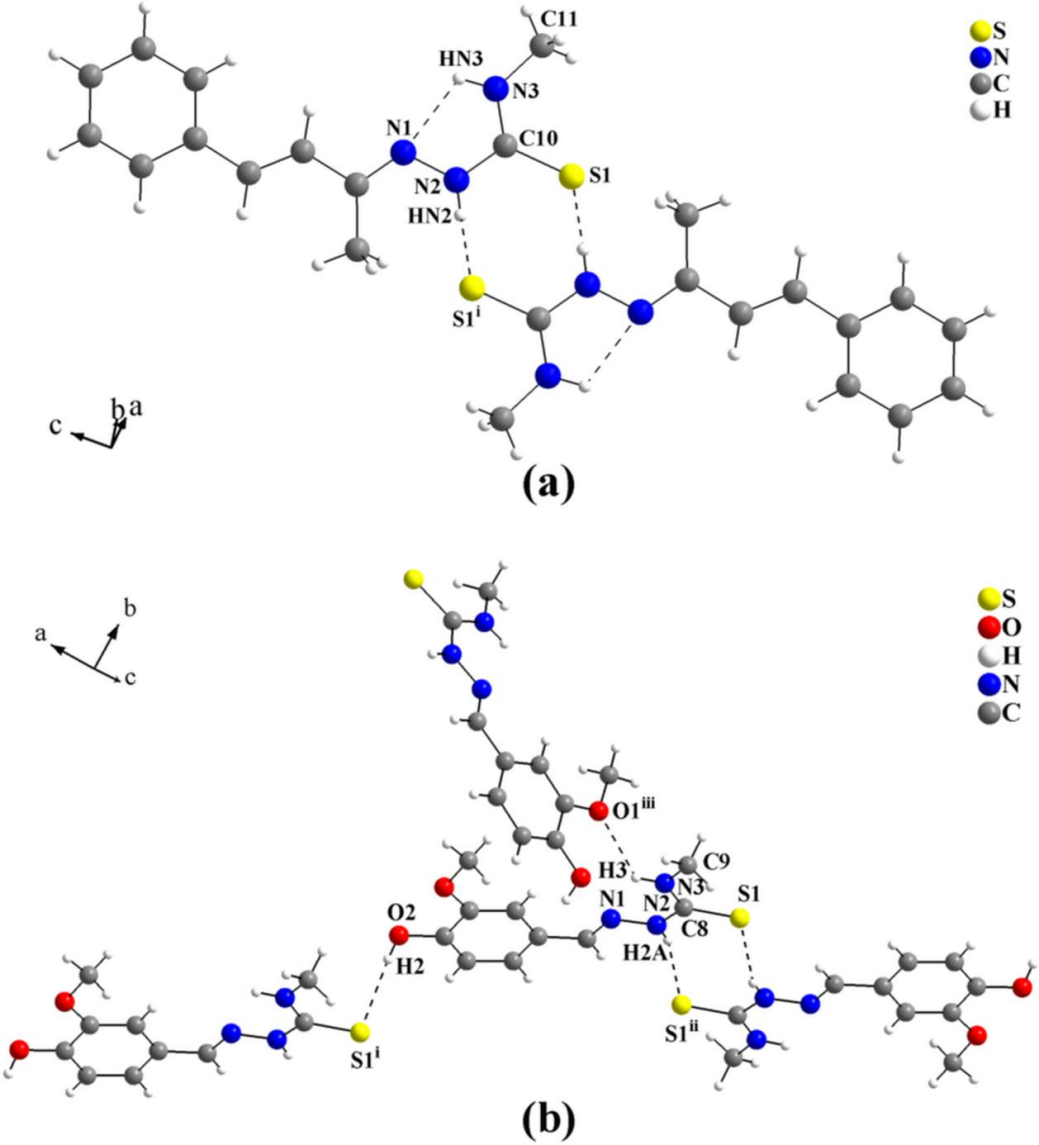
(*a*) Dimeric structure of the benzyl­ideneacetone 4-methyl­thio­semicarbazone compound (Rocha *et al.*, 2014 ▸). The mol­ecules are connected *via* pairs of centrosymmetric N—H⋯S inter­actions, with graph-set 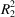 (8). [Symmetry code: (i) −*x* + 1, −*y*, −*z*.] and (*b*) section of the mol­ecular arrangement of the vanilline 4-methyl­thio­semicarbazone structure (Oliveira, Beck *et al.*, 2015 ▸). The mol­ecules are connected by pairs of centrosymmetric N—H⋯S inter­actions, with graph-set 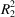 (8). The dimers are linked further by O—H⋯S and N—H⋯O inter­actions into a tape-like structure. Only the subunit of the supra­molecular arrangement is shown for clarity. [Symmetry codes: (i) *x* + 1, *y-*1, *z*;; (ii) −*x* − 2, −*y*, −*z*; (iii) -*x-*1, *y* + 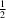 , −*z* − 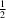 .]

The first example is the crystal structure of benzyl­ideneacetone 4-methyl­thio­semicarbazone (Rocha *et al.*, 2014 ▸). As a result of the steric effect of two methyl groups, one on the terminal N atom and other on the C atom attached to the thio­semicarbazone entity, dimer formation was favoured. The remaining N—H bond is involved in the N—H⋯N intra­molecular inter­action, with graph-set motif *S*(5). Thus, the mol­ecules are linked by N—H⋯S inter­actions, with graph-set motif 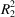 (8), into centrosymmetric dimers. For the graphical representation of the dimeric unit, see Fig. 7 ▸(*a*).

The second selected mol­ecule is the vanilline 4-methyl­thio­semicarbazone derivative (Oliveira, Beck *et al.*, 2015 ▸) in which the thio­semicarbazone entities are connected by N—H⋯S inter­actions, with graph-set motif 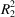 (8), into centrosymmetric dimers. The dimers are further linked through N—H⋯S and O—H⋯S inter­actions and can be considered subunits of a hydrogen-bonded tape-like supra­molecular arrangement. This is only possible because of the O atoms in the vanilline structure, see Fig. 7 ▸(*b*).

**Figure 8:**
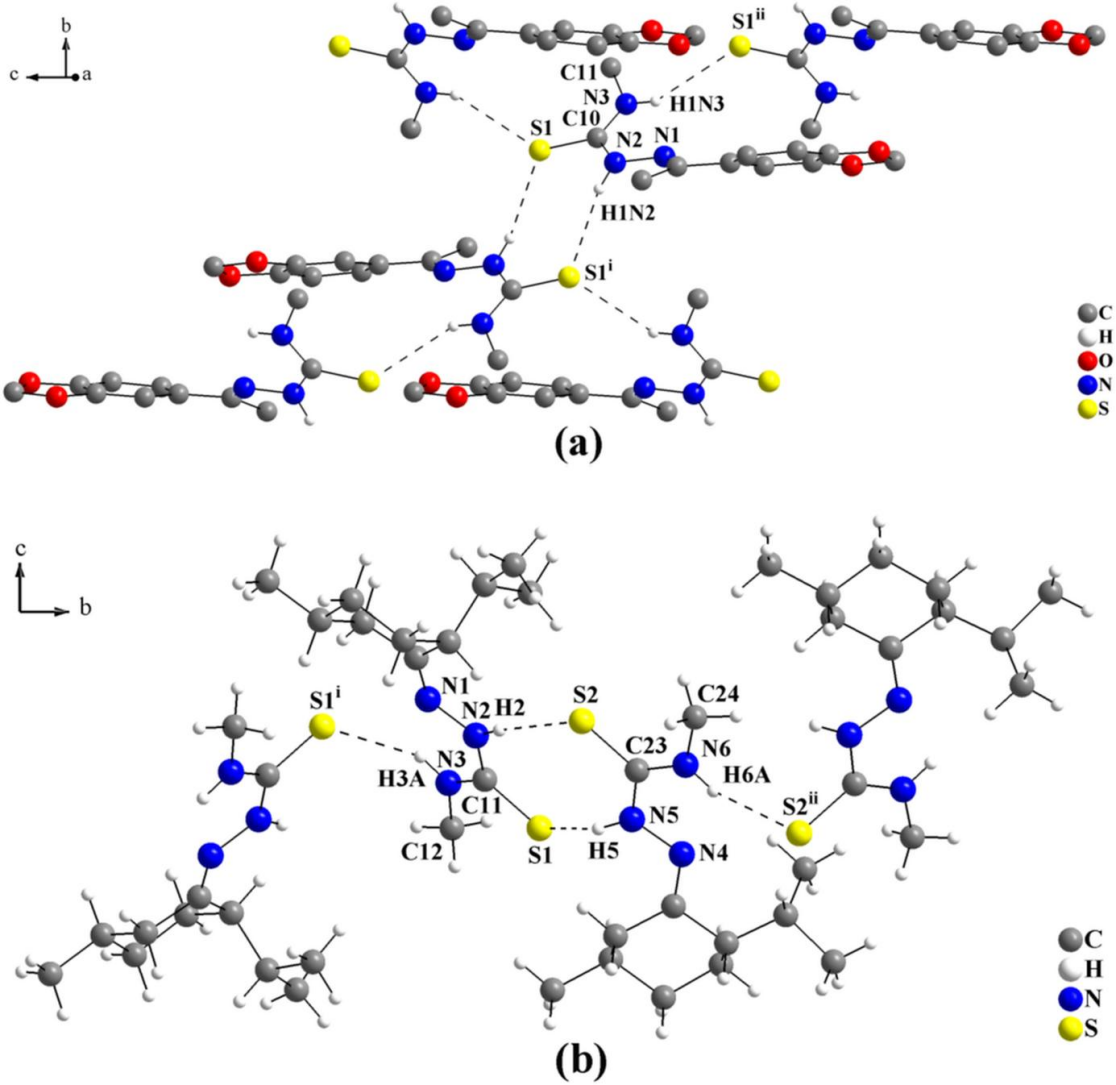
(*a*) Section of the mol­ecular arrangement of the 3′,4′-(methyl­enedi­oxy)aceto­phenone 4-methyl­thio­semicarbazone structure (Oliveira, Näther *et al.*, 2015 ▸). The mol­ecules are connected by pairs of centrosymmetric N—H⋯S inter­actions, with graph-set 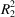 (8), and further linked by additional N—H⋯S inter­actions into a tape-like structure. H atoms were omitted for clarity and only the subunit of the supra­molecular arrangement is shown [Symmetry codes: (i) −*x* + 1, −*y* + 1, −*z* + 2; (ii) *x*, −*y* + 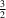 , *z* − 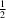 .] and (*b*) section of the mol­ecular arrangement of the (–)-menthone 4-methyl­thio­semicarbazone structure (Oliveira *et al.*, 2016 ▸). The mol­ecules are connected by pairs of N—H⋯S inter­actions, with graph-set 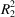 (8), into non-centrosymmetric dimers and further linked by additional N—H⋯S inter­actions, forming a tape-like structure. Only the subunit of the supra­molecular arrangement is shown for clarity [Symmetry codes: (i) −*x* + 1, *y* − 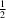 , −*z* + 1; (ii) −*x* + 2, *y* + 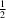 , −*z* + 1.]

A further example is 3′,4′-(methyl­enedi­oxy)aceto­phenone 4-methyl­thio­semicarbazone (Oliveira, Näther *et al.*, 2015 ▸). As mentioned above, the terminal methyl group decreases the dimensionality of the mol­ecular arrangement and the thio­semicarbazone entities are connected by pairs of centrosymmetric N—H⋯S inter­actions, with graph-set motifs 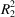 (8). A feature of the structural arrangement of this compound is that every thio­semicarbazone fragment bridges two other mol­ecules through N—H⋯S inter­actions in opposite directions, see Fig. 8 ▸(*a*).

Finally, the structure of (–)-menthone 4-methyl­thio­semicarbazone (Oliveira *et al.*, 2016 ▸) shows a non-centrosymmetric dimer, with the mol­ecules connected by pairs of N—H⋯S inter­actions, also with graph-set motif 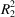 (8). A difference in this structure is the linking of the terminal N—H bonds between the mol­ecules through N—H⋯S inter­actions into a tape-like structure. For the dimeric subunit of the supra­molecular arrangement, see Fig. 8 ▸(*b*).

As observed for the title compound, pairs of N—H⋯S inter­molecular inter­actions with graph-set motif 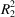 (8) are a remarkable feature for the crystal structure of thio­semicarbazone derivatives. The supra­molecular arrangement of the compounds depends on the structure of the substituents on the terminal N atom, as well as on the fragment attached to the first N atom.

## Synthesis and crystallization

The starting materials are commercially available and were used without further purification. The synthesis of the *cis*-jasmone 4-methyl­thio­semicarbazone derivative was adapted from previously reported procedures (Oliveira, Beck *et al.*, 2015 ▸; Orsoni *et al.*, 2020 ▸). A mixture of ethano­lic solutions of *cis*-jasmone (8 mmol in 50 ml) and 4-methyl­thio­semicarbazide (8 mmol in 50 ml) was catalysed with HCl and refluxed for 8 h. After cooling, the precipitated product was filtered off and washed with cold ethanol. Colourless single crystals suitable for X-ray diffraction were obtained from tetra­hydro­furan by slow evaporation of the solvent at room temperature.

## Refinement

Crystal data, data collection and structure refinement details are summarized in Table 3 ▸. There are two crystallographically independent mol­ecules in the asymmetric unit of the title compound and one of them, **JMTSC-2**, shows disorder over the chain of the *cis*-jasmone fragment, namely the C20, C21, C22, C23, H19*C* and H19*D* atoms (Fig. 1 ▸). These atoms were split over two positions, with the carbon atoms being *A*-labelled for the higher s.o.f. value positions and *B*-labelled for the lower [site-occupancy ratio = 0.821 (3):0.179 (3)]. The atom C19 is itself not disordered, but it is bound to C20*A* and C20*B*, and to get the best orientations for the C19—H bonds, the hydrogen atoms were disordered. Thus, H19*A* and H19*B* have the positions with higher s.o.f., while H19*C* and H19*D* have the positions with the lower. The EADP command was used to constrain the displacement parameters of the disordered carbon atoms.

The H atoms were treated by a mixture of constrained and independent refinement. The constrained H atoms were located in a difference-Fourier map, but were positioned with idealized geometry and refined using a riding model. For the C13H_3_, C23*A*H_3_, C23*B*H_3_ and C26H_3_ groups, the methyl H atoms were allowed to rotate but not to tip to best fit the experimental electron density, with *U*_iso_(H) = 1.5 *U*_eq_(C), and the C—H bonds were set to 0.96 Å. In an analogous manner, with *U*_iso_(H) = 1.2 *U*_eq_(C), for the C22*A*H_2_ and C22*B*H_2_ groups the C—H bond lengths were set to 0.97 Å and for the C20*A*H, C20*B*H, C21*A*H and C21*B*H, were set to 0.93 Å. In addition, the C19—H bonds were set to 0.97 Å. The remaining H atoms were refined freely.

## Supplementary Material

Crystal structure: contains datablock(s) I, publication_text. DOI: 10.1107/S2414314624000130/bt4146sup1.cif

Structure factors: contains datablock(s) I. DOI: 10.1107/S2414314624000130/bt4146Isup2.hkl

Supporting information file. DOI: 10.1107/S2414314624000130/bt4146Isup3.cml

CCDC reference: 2304272

Additional supporting information:  crystallographic information; 3D view; checkCIF report

## Figures and Tables

**Table 1:** Selected geometric parameters (Å, °) for the two crystallographically independent *cis*-jasmone 4-methyl­thio­semicarbazone mol­ecules, **JMTSC-1** and **JMTSC-2**

Compound	Atom chain	Torsion angle	Atom chain	Torsion angle
**JMTSC-1**	N1/N2/C11/N3	−1.2 (3)	C5/C6/C7/C8	114.6 (3)
**JMTSC-1**	N1/N2/C11/S1	178.55 (17)	C7/C8/C9/C10	128.0 (4)
**JMTSC-2**	N4/N5/C25/N6	0.8 (3)	C18/C19/C20*A*/C21*A*	139.9 (4)
**JMTSC-2**	N4/N5/C25/S2	−179.57 (16)	C18/C19/C20*B*/C21*B*	−117.6 (13)
			C20*A*/C21*A*/C22*A*/C23*A*	121.9 (4)
			C20*B*/C21*B*/C22*B*/C23*B*	−95 (4)
				
	Fragment	Max. deviation^*a*^	r.m.s.d.	Angle^*b*^
**JMTSC-1**	N1/N2/C11/S1/N3	−0.0115 (16) [N2]	0.0078	
**JMTSC-1**	C1—C5 ring	0.0130 (16) [C4]	0.0089	8.9 (1)
**JMTSC-2**	N4/N5/C25/S2/N6	0.0052 (14) [N5]	0.0031	
**JMTSC-2**	C14—C18 ring	0.0078 (16) [C17]	0.0054	6.3 (1)
				
	Bond lengths^*c*^	N—N	N—C	C=S
**JMTSC-1**		1.392 (3)	1.351 (3)	1.680 (2)
**JMTSC-2**		1.394 (2)	1.357 (3)	1.678 (2)

Notes: (*a*) The maximum deviation from the mean plane through the selected atoms; (*b*) angle to previous plane; (*c*) bond lengths for the N1/N2/C11/S1 and N4/N5/C25/S2 entities.

**Table 2:** Hydrogen-bond geometry (Å, °)

*D*—H⋯*A*	*D*—H	H⋯*A*	*D*⋯*A*	*D*—H⋯*A*
N2—H1⋯S1^i^	0.81 (3)	2.80 (3)	3.591 (2)	167 (2)
C2—H5*B*⋯S1^i^	0.97 (3)	2.90 (3)	3.457 (2)	117.4 (18)
N5—H3⋯S2^ii^	0.84 (3)	2.75 (3)	3.585 (2)	172 (2)
C15—H18*A*⋯S2^ii^	0.93 (2)	2.98 (2)	3.472 (2)	115.0 (17)

Symmetry codes: (i) 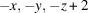 ; (ii) 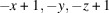 .

**Table 3:** Experimental details

Crystal data	
Chemical formula	C_13_H_21_N_3_S
*M*_r_	251.39
Crystal system, space group	Triclinic, *P* 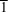
Temperature (K)	223
*a*, *b*, *c* (Å)	7.9583 (2), 11.2703 (2), 16.0080 (5)
α, β, γ (°)	83.0428 (18), 86.9392 (13), 76.5236 (18)
*V* (Å^3^)	1385.51 (6)
*Z*	4
Radiation type	Mo *K*α
μ (mm^−1^)	0.22
Crystal size (mm)	0.28 × 0.13 × 0.12

Data collection	
Diffractometer	Enraf–Nonius FR590 Kappa CCD
Absorption correction	Analytical (Alcock, 1970 ▸)
*T*_min_, *T*_max_	0.945, 0.978
No. of measured, independent and observed [*I* > 2σ(*I*)] reflections	23118, 6319, 3700
*R*_int_	0.056
(sin θ/λ)_max_ (Å^−1^)	0.650

Refinement	
*R*[*F* ^2^ > 2σ(*F* ^2^)], *wR*(*F* ^2^), *S*	0.052, 0.140, 1.03
No. of reflections	6319
No. of parameters	432
H-atom treatment	H atoms treated by a mixture of independent and constrained refinement
Δρ_max_, Δρ_min_ (e Å^−3^)	0.29, −0.24

Computer programs: *COLLECT* (Nonius, 1998 ▸), *HKL* *DENZO* and *SCALEPACK* (Otwinowski & Minor, 1997 ▸), *SIR92* (Altomare *et al.*, 1994 ▸), *SHELXL2018/3* (Sheldrick, 2015 ▸), *DIAMOND* (Brandenburg, 2006 ▸), *Crystal Explorer 3.1* (Wolff *et al.*, 2012 ▸), *WinGX* (Farrugia, 2012 ▸), *publCIF* (Westrip, 2010 ▸) and *enCIFer* (Allen *et al.*, 2004 ▸).

## full crystallographic data

### Crystal data

**Table:**

C_13_H_21_N_3_S	*Z* = 4
*M**_r_* = 251.39	*F*(000) = 544
Triclinic, *P*1	*D*_x_ = 1.205 Mg m^−^^3^
*a* = 7.9583 (2) Å	Mo *K*α radiation, λ = 0.71073 Å
*b* = 11.2703 (2) Å	Cell parameters from 22795 reflections
*c* = 16.0080 (5) Å	θ = 2.9–27.5°
α = 83.0428 (18)°	µ = 0.22 mm^−^^1^
β = 86.9392 (13)°	*T* = 223 K
γ = 76.5236 (18)°	Prism, colourless
*V* = 1385.51 (6) Å^3^	0.28 × 0.13 × 0.12 mm

### Data collection

**Table:**

Enraf–Nonius FR590 Kappa CCD diffractometer	6319 independent reflections
Radiation source: sealed X-ray tube, Enraf Nonius FR590	3700 reflections with *I* > 2σ(*I*)
Horizontally mounted graphite crystal monochromator	*R*_int_ = 0.056
Detector resolution: 9 pixels mm^-1^	θ_max_ = 27.5°, θ_min_ = 3.0°
CCD rotation images, thick slices, κ–goniostat scans	*h* = −10→10
Absorption correction: analytical (Alcock, 1970)	*k* = −14→14
*T*_min_ = 0.945, *T*_max_ = 0.978	*l* = −20→20
23118 measured reflections	

### Refinement

**Table:**

Refinement on *F*^2^	Primary atom site location: structure-invariant direct methods
Least-squares matrix: full	Secondary atom site location: difference Fourier map
*R*[*F*^2^ > 2σ(*F*^2^)] = 0.052	Hydrogen site location: mixed
*wR*(*F*^2^) = 0.140	H atoms treated by a mixture of independent and constrained refinement
*S* = 1.03	*w* = 1/[σ^2^(*F*_o_^2^) + (0.0556*P*)^2^ + 0.4261*P*] where *P* = (*F*_o_^2^ + 2*F*_c_^2^)/3
6319 reflections	(Δ/σ)_max_ = 0.001
432 parameters	Δρ_max_ = 0.29 e Å^−^^3^
0 restraints	Δρ_min_ = −0.24 e Å^−^^3^

### Special details

**Table:**

Geometry. All esds (except the esd in the dihedral angle between two l.s. planes) are estimated using the full covariance matrix. The cell esds are taken into account individually in the estimation of esds in distances, angles and torsion angles; correlations between esds in cell parameters are only used when they are defined by crystal symmetry. An approximate (isotropic) treatment of cell esds is used for estimating esds involving l.s. planes.

### Fractional atomic coordinates and isotropic or equivalent isotropic displacement parameters (Å^2^)

**Table:**

	*x*	*y*	*z*	*U*_iso_*/*U*_eq_	Occ. (<1)
C1	0.0353 (3)	0.1677 (2)	0.75329 (13)	0.0349 (5)	
C2	0.0002 (4)	0.0424 (2)	0.75249 (14)	0.0389 (5)	
C3	0.0007 (4)	0.0248 (2)	0.65901 (15)	0.0417 (6)	
C4	0.0398 (3)	0.1401 (2)	0.61351 (13)	0.0376 (5)	
C5	0.0551 (3)	0.2205 (2)	0.66637 (13)	0.0364 (5)	
C6	0.0898 (4)	0.3467 (2)	0.64395 (17)	0.0442 (6)	
C7	−0.0620 (4)	0.4410 (2)	0.60830 (17)	0.0486 (6)	
H7	−0.090 (3)	0.427 (3)	0.5551 (18)	0.067 (9)*	
C8	−0.1493 (4)	0.5392 (3)	0.6422 (2)	0.0583 (7)	
H8	−0.243 (3)	0.593 (2)	0.6108 (16)	0.055 (7)*	
C9	−0.1227 (6)	0.5789 (3)	0.7249 (2)	0.0773 (10)	
H9A	−0.009 (5)	0.516 (4)	0.750 (2)	0.124 (14)*	
H9B	−0.226 (5)	0.571 (3)	0.762 (2)	0.110 (13)*	
C10	−0.1051 (5)	0.7098 (3)	0.7187 (2)	0.0664 (9)	
H10A	−0.003 (5)	0.725 (3)	0.684 (2)	0.090 (11)*	
H10B	−0.095 (5)	0.735 (3)	0.771 (2)	0.103 (13)*	
H10C	−0.203 (5)	0.764 (3)	0.692 (2)	0.102 (12)*	
C11	0.0650 (3)	0.2114 (2)	0.96479 (14)	0.0410 (6)	
C12	0.0594 (4)	0.1544 (3)	0.52013 (16)	0.0502 (7)	
C13	0.1722 (4)	0.3754 (3)	1.01676 (17)	0.0605 (8)	
H13A	0.078825	0.394161	1.057058	0.091*	
H13B	0.202888	0.449897	0.992591	0.091*	
H13C	0.270256	0.321649	1.044276	0.091*	
H1	0.000 (3)	0.099 (3)	0.9014 (16)	0.055 (9)*	
H2	0.136 (4)	0.342 (3)	0.9011 (18)	0.062 (9)*	
H4A	−0.107 (3)	0.012 (2)	0.6423 (14)	0.043 (7)*	
H4B	0.087 (3)	−0.043 (2)	0.6446 (14)	0.041 (6)*	
H5A	−0.111 (4)	0.036 (2)	0.7836 (16)	0.062 (8)*	
H5B	0.083 (3)	−0.023 (2)	0.7831 (16)	0.055 (7)*	
H6A	0.132 (3)	0.375 (2)	0.6918 (14)	0.037 (6)*	
H6B	0.178 (4)	0.344 (2)	0.6043 (17)	0.058 (8)*	
H11A	−0.050 (4)	0.144 (3)	0.4942 (19)	0.078 (10)*	
H11B	0.151 (4)	0.097 (3)	0.5017 (18)	0.070 (9)*	
H11C	0.082 (4)	0.233 (3)	0.4981 (18)	0.078 (10)*	
N1	0.0527 (2)	0.22380 (17)	0.81644 (11)	0.0402 (5)	
N2	0.0324 (3)	0.1628 (2)	0.89595 (12)	0.0443 (5)	
N3	0.1185 (3)	0.3154 (2)	0.95063 (14)	0.0503 (6)	
S1	0.03760 (10)	0.14256 (6)	1.06201 (4)	0.0555 (2)	
C14	0.5379 (3)	0.16267 (19)	0.71740 (13)	0.0329 (5)	
C15	0.5078 (4)	0.0367 (2)	0.74221 (14)	0.0370 (5)	
C16	0.5116 (4)	0.0211 (2)	0.83872 (14)	0.0412 (6)	
C17	0.5457 (3)	0.1391 (2)	0.86107 (13)	0.0375 (5)	
C18	0.5580 (3)	0.21881 (19)	0.79294 (13)	0.0347 (5)	
C19	0.5811 (3)	0.3483 (2)	0.78856 (15)	0.0448 (6)	
H19A	0.643811	0.356753	0.837003	0.054*	0.821 (3)
H19B	0.647894	0.366698	0.738193	0.054*	0.821 (3)
H19C	0.685443	0.350346	0.754772	0.054*	0.179 (3)
H19D	0.486229	0.399219	0.755824	0.054*	0.179 (3)
C20A	0.4087 (5)	0.4364 (3)	0.7871 (2)	0.0532 (8)	0.821 (3)
H20A	0.329480	0.415962	0.754005	0.064*	0.821 (3)
C21A	0.3451 (5)	0.5388 (3)	0.8238 (2)	0.0569 (9)	0.821 (3)
H21A	0.229448	0.576497	0.815022	0.068*	0.821 (3)
C22A	0.4385 (6)	0.5964 (3)	0.8758 (2)	0.0621 (10)	0.821 (3)
H22A	0.557592	0.550356	0.879680	0.075*	0.821 (3)
H22B	0.387225	0.595899	0.932190	0.075*	0.821 (3)
C23A	0.4333 (6)	0.7287 (3)	0.8386 (3)	0.0612 (11)	0.821 (3)
H23A	0.474681	0.729616	0.781112	0.092*	0.821 (3)
H23B	0.505314	0.763050	0.870457	0.092*	0.821 (3)
H23C	0.316591	0.776477	0.840756	0.092*	0.821 (3)
C20B	0.592 (2)	0.4084 (12)	0.8561 (9)	0.0532 (8)	0.179 (3)
H20B	0.682753	0.377545	0.892534	0.064*	0.179 (3)
C21B	0.479 (2)	0.5069 (15)	0.8714 (11)	0.0569 (9)	0.179 (3)
H21B	0.516842	0.521502	0.922436	0.068*	0.179 (3)
C22B	0.319 (3)	0.6156 (17)	0.8557 (11)	0.0621 (10)	0.179 (3)
H22C	0.239982	0.592198	0.820463	0.075*	0.179 (3)
H22D	0.260444	0.632136	0.909131	0.075*	0.179 (3)
C23B	0.366 (3)	0.728 (2)	0.8152 (14)	0.0612 (11)	0.179 (3)
H23D	0.363076	0.783573	0.856476	0.092*	0.179 (3)
H23E	0.284292	0.766305	0.772294	0.092*	0.179 (3)
H23F	0.479632	0.707879	0.790243	0.092*	0.179 (3)
C24	0.5633 (5)	0.1554 (3)	0.95104 (15)	0.0526 (7)	
C25	0.5613 (3)	0.2125 (2)	0.49665 (13)	0.0354 (5)	
C26	0.6433 (4)	0.3925 (2)	0.41676 (15)	0.0529 (7)	
H26A	0.739134	0.344372	0.387775	0.079*	
H26B	0.673244	0.465559	0.430318	0.079*	
H26C	0.544756	0.414694	0.381308	0.079*	
H3	0.508 (3)	0.089 (3)	0.5819 (16)	0.052 (8)*	
H4	0.611 (3)	0.348 (2)	0.5401 (16)	0.048 (7)*	
H17A	0.599 (3)	−0.050 (2)	0.8611 (15)	0.053 (7)*	
H17B	0.401 (3)	0.004 (2)	0.8647 (15)	0.050 (7)*	
H18A	0.590 (3)	−0.023 (2)	0.7182 (14)	0.044 (7)*	
H18B	0.398 (3)	0.032 (2)	0.7211 (15)	0.048 (7)*	
H24A	0.573 (4)	0.238 (3)	0.960 (2)	0.095 (11)*	
H24B	0.459 (4)	0.138 (3)	0.9862 (18)	0.070 (9)*	
H24C	0.662 (4)	0.096 (3)	0.9751 (19)	0.078 (10)*	
N4	0.5513 (2)	0.22004 (16)	0.64328 (11)	0.0370 (4)	
N5	0.5353 (3)	0.15779 (19)	0.57504 (11)	0.0384 (5)	
N6	0.6022 (3)	0.32130 (18)	0.49376 (13)	0.0453 (5)	
S2	0.54338 (9)	0.14458 (6)	0.41094 (3)	0.04489 (19)	

### Atomic displacement parameters (Å^2^)

**Table:**

	*U*^11^	*U*^22^	*U*^33^	*U*^12^	*U*^13^	*U*^23^
C1	0.0328 (13)	0.0353 (12)	0.0357 (12)	−0.0050 (10)	−0.0016 (9)	−0.0056 (9)
C2	0.0442 (15)	0.0375 (13)	0.0337 (12)	−0.0094 (11)	0.0011 (11)	−0.0002 (10)
C3	0.0490 (16)	0.0385 (14)	0.0375 (13)	−0.0090 (12)	0.0019 (11)	−0.0076 (10)
C4	0.0383 (13)	0.0390 (13)	0.0335 (12)	−0.0059 (10)	0.0031 (10)	−0.0035 (9)
C5	0.0373 (13)	0.0366 (13)	0.0340 (12)	−0.0063 (10)	−0.0003 (9)	−0.0028 (9)
C6	0.0495 (16)	0.0426 (14)	0.0425 (15)	−0.0161 (12)	−0.0001 (13)	−0.0023 (11)
C7	0.0600 (18)	0.0396 (15)	0.0477 (16)	−0.0185 (13)	−0.0049 (13)	0.0053 (11)
C8	0.0541 (18)	0.0418 (16)	0.075 (2)	−0.0109 (13)	−0.0013 (15)	0.0083 (14)
C9	0.111 (3)	0.0500 (19)	0.064 (2)	−0.0098 (19)	0.033 (2)	−0.0092 (15)
C10	0.078 (2)	0.0518 (19)	0.067 (2)	−0.0093 (17)	0.0050 (19)	−0.0087 (15)
C11	0.0462 (15)	0.0418 (14)	0.0355 (13)	−0.0086 (11)	−0.0052 (10)	−0.0072 (10)
C12	0.063 (2)	0.0497 (17)	0.0366 (14)	−0.0105 (15)	0.0044 (13)	−0.0067 (12)
C13	0.083 (2)	0.0542 (17)	0.0518 (16)	−0.0235 (15)	−0.0136 (14)	−0.0132 (13)
N1	0.0460 (12)	0.0425 (11)	0.0324 (10)	−0.0114 (9)	−0.0009 (8)	−0.0029 (8)
N2	0.0604 (14)	0.0447 (13)	0.0321 (11)	−0.0210 (11)	−0.0027 (9)	−0.0035 (9)
N3	0.0715 (16)	0.0471 (13)	0.0372 (13)	−0.0233 (11)	−0.0059 (11)	−0.0036 (10)
S1	0.0842 (5)	0.0544 (4)	0.0327 (3)	−0.0261 (4)	−0.0033 (3)	−0.0034 (3)
C14	0.0371 (13)	0.0325 (12)	0.0292 (11)	−0.0085 (10)	0.0009 (9)	−0.0039 (9)
C15	0.0450 (15)	0.0325 (13)	0.0335 (12)	−0.0087 (11)	0.0003 (11)	−0.0045 (9)
C16	0.0585 (17)	0.0331 (13)	0.0319 (12)	−0.0119 (12)	0.0020 (12)	−0.0014 (9)
C17	0.0463 (14)	0.0353 (12)	0.0307 (12)	−0.0085 (10)	0.0020 (10)	−0.0064 (9)
C18	0.0404 (13)	0.0338 (12)	0.0315 (11)	−0.0110 (10)	0.0006 (9)	−0.0057 (9)
C19	0.0614 (17)	0.0410 (14)	0.0381 (13)	−0.0233 (12)	−0.0009 (11)	−0.0059 (10)
C20A	0.071 (2)	0.0345 (16)	0.0559 (19)	−0.0156 (15)	−0.0056 (16)	−0.0041 (13)
C21A	0.054 (2)	0.0441 (19)	0.072 (2)	−0.0135 (16)	−0.0031 (16)	−0.0019 (16)
C22A	0.089 (3)	0.052 (2)	0.049 (2)	−0.024 (2)	−0.004 (2)	−0.0035 (15)
C23A	0.092 (4)	0.0384 (17)	0.057 (3)	−0.023 (2)	−0.008 (2)	−0.0053 (17)
C20B	0.071 (2)	0.0345 (16)	0.0559 (19)	−0.0156 (15)	−0.0056 (16)	−0.0041 (13)
C21B	0.054 (2)	0.0441 (19)	0.072 (2)	−0.0135 (16)	−0.0031 (16)	−0.0019 (16)
C22B	0.089 (3)	0.052 (2)	0.049 (2)	−0.024 (2)	−0.004 (2)	−0.0035 (15)
C23B	0.092 (4)	0.0384 (17)	0.057 (3)	−0.023 (2)	−0.008 (2)	−0.0053 (17)
C24	0.079 (2)	0.0511 (18)	0.0284 (13)	−0.0152 (16)	−0.0032 (14)	−0.0050 (11)
C25	0.0385 (13)	0.0341 (12)	0.0324 (12)	−0.0075 (10)	0.0000 (9)	−0.0013 (9)
C26	0.0690 (18)	0.0466 (15)	0.0443 (14)	−0.0222 (13)	0.0037 (13)	0.0056 (11)
N4	0.0457 (12)	0.0375 (11)	0.0302 (10)	−0.0129 (9)	−0.0012 (8)	−0.0067 (8)
N5	0.0550 (13)	0.0338 (11)	0.0287 (10)	−0.0147 (10)	0.0006 (8)	−0.0044 (8)
N6	0.0667 (15)	0.0414 (12)	0.0314 (11)	−0.0209 (10)	0.0021 (10)	−0.0027 (9)
S2	0.0637 (4)	0.0434 (4)	0.0286 (3)	−0.0142 (3)	−0.0008 (3)	−0.0043 (2)

### Geometric parameters (Å, º)

**Table:**

C1—N1	1.285 (3)	C16—H17A	0.98 (3)
C1—C5	1.461 (3)	C16—H17B	1.00 (3)
C1—C2	1.504 (3)	C17—C18	1.341 (3)
C2—C3	1.533 (3)	C17—C24	1.492 (3)
C2—H5A	1.00 (3)	C18—C19	1.506 (3)
C2—H5B	0.97 (3)	C19—C20B	1.359 (14)
C3—C4	1.500 (3)	C19—C20A	1.493 (4)
C3—H4A	0.96 (2)	C19—H19A	0.9700
C3—H4B	0.95 (2)	C19—H19B	0.9700
C4—C5	1.342 (3)	C19—H19C	0.9700
C4—C12	1.488 (3)	C19—H19D	0.9700
C5—C6	1.509 (3)	C20A—C21A	1.339 (5)
C6—C7	1.498 (4)	C20A—H20A	0.9300
C6—H6A	0.97 (2)	C21A—C22A	1.445 (5)
C6—H6B	0.92 (3)	C21A—H21A	0.9300
C7—C8	1.323 (4)	C22A—C23A	1.530 (5)
C7—H7	0.93 (3)	C22A—H22A	0.9700
C8—C9	1.488 (5)	C22A—H22B	0.9700
C8—H8	0.96 (3)	C23A—H23A	0.9600
C9—C10	1.505 (5)	C23A—H23B	0.9600
C9—H9A	1.07 (4)	C23A—H23C	0.9600
C9—H9B	1.00 (4)	C20B—C21B	1.29 (2)
C10—H10A	1.00 (4)	C20B—H20B	0.9300
C10—H10B	0.93 (4)	C21B—C22B	1.56 (3)
C10—H10C	0.96 (4)	C21B—H21B	0.9300
C11—N3	1.329 (3)	C22B—C23B	1.47 (3)
C11—N2	1.351 (3)	C22B—H22C	0.9700
C11—S1	1.680 (2)	C22B—H22D	0.9700
C12—H11A	1.02 (3)	C23B—H23D	0.9600
C12—H11B	0.91 (3)	C23B—H23E	0.9600
C12—H11C	0.96 (3)	C23B—H23F	0.9600
C13—N3	1.455 (3)	C24—H24A	0.97 (4)
C13—H13A	0.9600	C24—H24B	1.02 (3)
C13—H13B	0.9600	C24—H24C	0.97 (3)
C13—H13C	0.9600	C25—N6	1.335 (3)
N1—N2	1.392 (3)	C25—N5	1.357 (3)
N2—H1	0.81 (3)	C25—S2	1.678 (2)
N3—H2	0.83 (3)	C26—N6	1.453 (3)
C14—N4	1.292 (3)	C26—H26A	0.9600
C14—C18	1.463 (3)	C26—H26B	0.9600
C14—C15	1.498 (3)	C26—H26C	0.9600
C15—C16	1.535 (3)	N4—N5	1.394 (2)
C15—H18A	0.93 (2)	N5—H3	0.84 (3)
C15—H18B	0.97 (3)	N6—H4	0.85 (3)
C16—C17	1.505 (3)		
			
N1—C1—C5	122.2 (2)	C17—C16—H17B	112.7 (14)
N1—C1—C2	129.2 (2)	C15—C16—H17B	111.6 (14)
C5—C1—C2	108.56 (18)	H17A—C16—H17B	104 (2)
C1—C2—C3	104.79 (19)	C18—C17—C24	127.8 (2)
C1—C2—H5A	111.1 (15)	C18—C17—C16	112.35 (19)
C3—C2—H5A	113.8 (15)	C24—C17—C16	119.8 (2)
C1—C2—H5B	114.0 (16)	C17—C18—C14	109.11 (19)
C3—C2—H5B	111.5 (15)	C17—C18—C19	128.8 (2)
H5A—C2—H5B	102 (2)	C14—C18—C19	122.04 (19)
C4—C3—C2	104.5 (2)	C20B—C19—C18	125.2 (6)
C4—C3—H4A	111.7 (14)	C20A—C19—C18	109.9 (2)
C2—C3—H4A	112.7 (14)	C20A—C19—H19A	109.7
C4—C3—H4B	108.8 (14)	C18—C19—H19A	109.7
C2—C3—H4B	112.6 (14)	C20A—C19—H19B	109.7
H4A—C3—H4B	107 (2)	C18—C19—H19B	109.7
C5—C4—C12	127.4 (2)	H19A—C19—H19B	108.2
C5—C4—C3	112.30 (19)	C20B—C19—H19C	106.0
C12—C4—C3	120.3 (2)	C18—C19—H19C	106.0
C4—C5—C1	109.8 (2)	C20B—C19—H19D	106.0
C4—C5—C6	127.5 (2)	C18—C19—H19D	106.0
C1—C5—C6	122.7 (2)	H19C—C19—H19D	106.3
C7—C6—C5	114.0 (2)	C21A—C20A—C19	133.1 (3)
C7—C6—H6A	110.1 (13)	C21A—C20A—H20A	113.5
C5—C6—H6A	111.0 (13)	C19—C20A—H20A	113.5
C7—C6—H6B	107.0 (16)	C20A—C21A—C22A	126.4 (4)
C5—C6—H6B	110.0 (17)	C20A—C21A—H21A	116.8
H6A—C6—H6B	104 (2)	C22A—C21A—H21A	116.8
C8—C7—C6	127.3 (3)	C21A—C22A—C23A	110.4 (3)
C8—C7—H7	119.7 (17)	C21A—C22A—H22A	109.6
C6—C7—H7	112.9 (17)	C23A—C22A—H22A	109.6
C7—C8—C9	128.0 (3)	C21A—C22A—H22B	109.6
C7—C8—H8	117.7 (16)	C23A—C22A—H22B	109.6
C9—C8—H8	114.3 (15)	H22A—C22A—H22B	108.1
C8—C9—C10	113.3 (3)	C22A—C23A—H23A	109.5
C8—C9—H9A	106 (2)	C22A—C23A—H23B	109.5
C10—C9—H9A	112 (2)	H23A—C23A—H23B	109.5
C8—C9—H9B	107 (2)	C22A—C23A—H23C	109.5
C10—C9—H9B	109 (2)	H23A—C23A—H23C	109.5
H9A—C9—H9B	110 (3)	H23B—C23A—H23C	109.5
C9—C10—H10A	113.2 (19)	C21B—C20B—C19	122.2 (14)
C9—C10—H10B	113 (2)	C21B—C20B—H20B	118.9
H10A—C10—H10B	107 (3)	C19—C20B—H20B	118.9
C9—C10—H10C	110 (2)	C20B—C21B—C22B	157.0 (17)
H10A—C10—H10C	105 (3)	C20B—C21B—H21B	101.5
H10B—C10—H10C	108 (3)	C22B—C21B—H21B	101.5
N3—C11—N2	116.2 (2)	C23B—C22B—C21B	112.7 (18)
N3—C11—S1	122.86 (18)	C23B—C22B—H22C	109.0
N2—C11—S1	120.97 (19)	C21B—C22B—H22C	109.0
C4—C12—H11A	109.5 (17)	C23B—C22B—H22D	109.0
C4—C12—H11B	111.9 (18)	C21B—C22B—H22D	109.0
H11A—C12—H11B	108 (2)	H22C—C22B—H22D	107.8
C4—C12—H11C	112.6 (18)	C22B—C23B—H23D	109.5
H11A—C12—H11C	110 (2)	C22B—C23B—H23E	109.5
H11B—C12—H11C	105 (3)	H23D—C23B—H23E	109.5
N3—C13—H13A	109.5	C22B—C23B—H23F	109.5
N3—C13—H13B	109.5	H23D—C23B—H23F	109.5
H13A—C13—H13B	109.5	H23E—C23B—H23F	109.5
N3—C13—H13C	109.5	C17—C24—H24A	114 (2)
H13A—C13—H13C	109.5	C17—C24—H24B	110.5 (16)
H13B—C13—H13C	109.5	H24A—C24—H24B	108 (3)
C1—N1—N2	116.5 (2)	C17—C24—H24C	110.6 (18)
C11—N2—N1	119.4 (2)	H24A—C24—H24C	109 (3)
C11—N2—H1	119.8 (19)	H24B—C24—H24C	105 (2)
N1—N2—H1	120.8 (19)	N6—C25—N5	115.3 (2)
C11—N3—C13	123.6 (2)	N6—C25—S2	123.71 (17)
C11—N3—H2	118 (2)	N5—C25—S2	120.96 (17)
C13—N3—H2	118 (2)	N6—C26—H26A	109.5
N4—C14—C18	120.8 (2)	N6—C26—H26B	109.5
N4—C14—C15	129.58 (19)	H26A—C26—H26B	109.5
C18—C14—C15	109.57 (18)	N6—C26—H26C	109.5
C14—C15—C16	104.14 (19)	H26A—C26—H26C	109.5
C14—C15—H18A	112.0 (15)	H26B—C26—H26C	109.5
C16—C15—H18A	112.9 (14)	C14—N4—N5	116.71 (18)
C14—C15—H18B	109.4 (14)	C25—N5—N4	117.8 (2)
C16—C15—H18B	112.9 (14)	C25—N5—H3	120.8 (17)
H18A—C15—H18B	106 (2)	N4—N5—H3	121.4 (17)
C17—C16—C15	104.81 (19)	C25—N6—C26	124.3 (2)
C17—C16—H17A	111.6 (15)	C25—N6—H4	117.6 (17)
C15—C16—H17A	112.8 (14)	C26—N6—H4	118.0 (18)
			
N1—C1—C2—C3	−177.8 (2)	C15—C16—C17—C18	1.3 (3)
C5—C1—C2—C3	0.0 (3)	C15—C16—C17—C24	−178.2 (2)
C1—C2—C3—C4	1.2 (3)	C24—C17—C18—C14	178.0 (2)
C2—C3—C4—C5	−2.2 (3)	C16—C17—C18—C14	−1.4 (3)
C2—C3—C4—C12	177.1 (2)	C24—C17—C18—C19	−4.4 (4)
C12—C4—C5—C1	−176.9 (2)	C16—C17—C18—C19	176.2 (2)
C3—C4—C5—C1	2.3 (3)	N4—C14—C18—C17	−177.4 (2)
C12—C4—C5—C6	2.3 (4)	C15—C14—C18—C17	0.9 (3)
C3—C4—C5—C6	−178.5 (2)	N4—C14—C18—C19	4.8 (3)
N1—C1—C5—C4	176.6 (2)	C15—C14—C18—C19	−176.9 (2)
C2—C1—C5—C4	−1.4 (3)	C17—C18—C19—C20B	−0.8 (9)
N1—C1—C5—C6	−2.7 (3)	C14—C18—C19—C20B	176.5 (9)
C2—C1—C5—C6	179.3 (2)	C17—C18—C19—C20A	−91.4 (3)
C4—C5—C6—C7	73.9 (3)	C14—C18—C19—C20A	85.9 (3)
C1—C5—C6—C7	−107.0 (3)	C18—C19—C20A—C21A	139.9 (4)
C5—C6—C7—C8	114.6 (3)	C19—C20A—C21A—C22A	2.8 (6)
C6—C7—C8—C9	−2.1 (5)	C20A—C21A—C22A—C23A	121.9 (4)
C7—C8—C9—C10	128.0 (4)	C18—C19—C20B—C21B	−117.6 (13)
C5—C1—N1—N2	−179.33 (19)	C19—C20B—C21B—C22B	−4 (5)
C2—C1—N1—N2	−1.8 (4)	C20B—C21B—C22B—C23B	−95 (4)
N3—C11—N2—N1	−1.2 (3)	C18—C14—N4—N5	178.16 (19)
S1—C11—N2—N1	178.55 (17)	C15—C14—N4—N5	0.2 (4)
C1—N1—N2—C11	173.8 (2)	N6—C25—N5—N4	0.8 (3)
N2—C11—N3—C13	−175.1 (2)	S2—C25—N5—N4	−179.57 (16)
S1—C11—N3—C13	5.1 (4)	C14—N4—N5—C25	−175.3 (2)
N4—C14—C15—C16	178.1 (2)	N5—C25—N6—C26	176.9 (2)
C18—C14—C15—C16	−0.1 (3)	S2—C25—N6—C26	−2.7 (4)
C14—C15—C16—C17	−0.6 (3)		

### Hydrogen-bond geometry (Å, º)

**Table:**

*D*—H···*A*	*D*—H	H···*A*	*D*···*A*	*D*—H···*A*
N2—H1···S1^i^	0.81 (3)	2.80 (3)	3.591 (2)	167 (2)
C2—H5*B*···S1^i^	0.97 (3)	2.90 (3)	3.457 (2)	117.4 (18)
N5—H3···S2^ii^	0.84 (3)	2.75 (3)	3.585 (2)	172 (2)
C15—H18*A*···S2^ii^	0.93 (2)	2.98 (2)	3.472 (2)	115.0 (17)

Symmetry codes: (i) −*x*, −*y*, −*z*+2; (ii) −*x*+1, −*y*, −*z*+1.
